# Acute exercise and high-glucose ingestion elicit dynamic and individualized responses in systemic markers of redox homeostasis

**DOI:** 10.3389/fimmu.2023.1127088

**Published:** 2023-03-30

**Authors:** Hannah J. Thomas, Teddy Ang, Dale J. Morrison, Michelle A. Keske, Lewan Parker

**Affiliations:** ^1^ Institute for Physical Activity and Nutrition (IPAN), School of Exercise and Nutrition Sciences, Deakin University, Geelong, VIC, Australia; ^2^ Department of Medicine, University of Melbourne, Melbourne, VIC, Australia

**Keywords:** oxidative stress, antioxidant, hyperglycemia, acute exercise, non-responder, redox, personalized, biomarker

## Abstract

**Background:**

Biomarkers of oxidation-reduction (redox) homeostasis are commonly measured in human blood to assess whether certain stimuli (e.g., high-glucose ingestion or acute exercise) lead to a state of oxidative distress (detrimental to health) or oxidative eustress (beneficial to health). Emerging research indicates that redox responses are likely to be highly individualized, yet few studies report individual responses. Furthermore, the effects of complex redox stimuli (e.g., high-glucose-ingestion after exercise) on redox homeostasis remains unclear. We investigated the effect of acute exercise (oxidative eustress), high-glucose ingestion (oxidative distress), and high-glucose ingestion after exercise (both oxidative eu/distress), on commonly measured redox biomarkers in serum/plasma.

**Methods:**

In a randomized crossover fashion, eight healthy men (age: 28 ± 4 years; BMI: 24.5 ± 1.5 kg/m^2^ [mean ± SD]) completed two separate testing conditions; 1) consumption of a high-glucose mixed-nutrient meal (45% carbohydrate [1.1 g glucose.kg^-1^], 20% protein, and 35% fat) at rest (control trial), and 2) consumption of the same meal 3 h and 24 h after 1 h of moderate-intensity cycling exercise (exercise trial). Plasma and serum were analyzed for an array of commonly studied redox biomarkers.

**Results:**

Oxidative stress and antioxidant defense markers (hydrogen peroxide, 8-isoprostanes, catalase, superoxide dismutase, and nitrate levels) increased immediately after exercise (p < 0.05), whereas nitric oxide activity and thiobarbituric acid reactive substances (TBARS) remained similar to baseline (p > 0.118). Nitric oxide activity and nitrate levels decreased at 3 h post-exercise compared to pre-exercise baseline levels. Depending on when the high-glucose mixed nutrient meal was ingested and the postprandial timepoint investigated, oxidative stress and antioxidant defense biomarkers either increased (hydrogen peroxide, TBARS, and superoxide dismutase), decreased (hydrogen peroxide, 8-isoprostanes, superoxide dismutase, nitric oxide activity, nitrate, and nitrite), or remained similar to pre-meal baseline levels (hydrogen peroxide, 8-isoprostanes, TBARS, catalase, superoxide dismutase and nitrite). Redox responses exhibited large inter-individual variability in the magnitude and/or direction of responses.

**Conclusion:**

Findings highlight the necessity to interpret redox biomarkers in the context of the individual, biomarker measured, and stimuli observed. Individual redox responsiveness may be of physiological relevance and should be explored as a potential means to inform personalized redox intervention.

## 1 Introduction

Reactive oxygen species (ROS) are unequivocally involved in physiological and pathological processes that regulate both human health and disease (1–6). For example, hyperglycemia (elevated blood glucose levels) leads to ROS production which contributes to the development of insulin resistance, type 2 diabetes, and other cardiometabolic diseases (5, 7–10). On the other hand, ROS are also required for glucose cell transport in response to insulin-stimulation and skeletal muscle contraction/exercise (1–4, 11–25). The concept that ROS can be “good” or “bad” depending on the context has led to the emerging use of the terms “oxidative eustress” and “oxidative distress” (13, 26, 27).

Oxidation-reduction (redox) biomarkers are commonly measured in blood or muscle tissue to study redox stimuli and to identify conditions of oxidative distress (e.g., glycemia-induced oxidative stress) or oxidative eustress (e.g., exercise-induced oxidative stress) (13, 14, 28). Commonly researched biomarkers include catalase activity, superoxide activity (SOD), thiobarbituric acid reactive substances (TBARS), 8-isoprostanes, nitric oxide activity (NOx), and hydrogen peroxide (H_2_O_2_). Many studies rely upon a single biomarker to inform upon whether a stimulus leads to oxidative eustress or distress. However, this can be problematic as not all redox biomarkers respond similarly to redox stimuli, for example exercise (29, 30), which can hamper overall interpretation of the findings. Furthermore, research has yet to investigate the response of commonly measured redox biomarkers following more complex stimuli (e.g., high-glucose ingestion after exercise) that is likely to involve varying degrees of both oxidative eustress and distress.

Characterizing the human redox response to certain stimuli (e.g., glycemia or exercise) is critically important for the development of new and more effective redox interventions that can target both oxidative eustress and distress. However, emerging research has reported that redox responses to certain stimuli are likely to be highly individualized (6, 31, 32). Margaritelis, Theodorou (6) stratified healthy individuals based on the degree of exercise-induced oxidative stress and revealed that individuals who experienced greater oxidative stress after exercise also exhibited greater exercise-induced training health adaptations including exercise capacity and performance (6). This concept of individual responsiveness to exercise is not surprising given the growing evidence of response variability in adaptations to exercise training (33–38). Our research group and others have also reported that the effectiveness of antioxidant treatment in targeting oxidative distress is likely to be highly individualized, and therefore requires a personalized antioxidant treatment approach (13, 39). However, to our knowledge, research has yet to examine individual responsiveness to meal ingestion (oxidative distress), exercise (oxidative eustress), and when exercise is performed prior to meal ingestion (oxidative eu/distress), in a randomized crossover design. Demonstrating the possibility of individual responsiveness in the context of redox homeostasis is a critical first step to informing future personalized redox interventions that can target both oxidative distress and eustress (13, 39, 40).

In this study we aimed to 1) explore the use of systemic markers of redox homeostasis to identify conditions of oxidative eustress and/or distress in humans, and 2) preliminarily investigate the extent of individual responsiveness to various redox stimuli. To achieve these aims, we compared the effects of acute exercise, high-glucose mixed-nutrient meal ingestion, and exercise prior to meal ingestion, on an array of commonly studied redox biomarkers in young healthy men.

## 2 Methods

### 2.1 Study overview

Serum and plasma samples from eight participants who participated in a previously published study were analyzed for various markers of redox homeostasis (41). Details of the study design and a protocol figure can be found in the previously published manuscript (41). Participants were young (age: 28 [25, 30] years [median ± IQR]) and apparently healthy (BMI: 24.3 [23.6, 25.5] kg/m^2^). Participants underwent a screening, familiarization, and exercise test session, followed by a control trial (meal ingested at rest) and an exercise trial (meal ingested both 3 h and 24 h post-exercise), performed in a randomized order with a minimum one-week between trials. The control trial involved the ingestion of a high-glucose mixed-nutrient meal at rest. The exercise trial spanned two testing visits and involved ingestion of the same meal at 3 h and 24 h after an acute session of aerobic cycling exercise. Participants were asked to abstain from caffeine (24 h), moderate to vigorous activity (48 h), and alcohol (24 h) prior to each testing session. Participants’ diet was recorded for 24 h prior to their first testing session which was then replicated prior to their subsequent testing session including the replication of afternoon snacks/meals in between the 3 h and 24 h post-exercise meals. The study was approved by the Deakin University Human Research Ethics Committee (DUHREC: 2018-010) and adhered to the standards set by the Declaration of Helsinki except for being registered in a publicly available database.

### 2.2 Pre-screening and cardiopulmonary exercise test

Participants were screened for eligibility *via* medical history and physical activity questionnaires, and resting measurements of blood pressure, heart rate, height, and weight. Participants were excluded if they had a previous history of cardiometabolic disease (e.g., diabetes or cardiovascular disease) or smoking, were taking medications or vitamins that may have affected vascular or glucoregulatory measures, or had musculoskeletal or other conditions that prevented daily activity. Eligible participants attended a familiarization and exercise test session where they were familiarized with the study protocols and equipment. Participants then underwent a maximal cardiopulmonary exercise test on a cycle ergometer (Lode Excalibur Sport, Lode, Groningen, The Netherlands) to measure peak aerobic capacity (VO_2peak_) and Watt max (W_max_). The cardiopulmonary exercise test started at 50 Watts and increased by 25 Watts every three minutes for the first three stages and then by 25 Watts every one minute until volitional exhaustion. A Quark metabolic cart with face mask (Quark RMR Gas Analyzer, Cosmed, Italy) was use for indirect calorimetry measures, as previously described (41).

### 2.3 Control trial (rest-control meal)

Participants arrived in the laboratory after an overnight fast and a cannula was inserted into an antecubital fossa vein. After 30 min of rest on a bed (supine) a baseline blood sample was taken. Participants then consumed the mixed-nutrient meal (10 kcal.kg^-1^; 45% carbohydrate, 20% protein, and 35% fat) containing eggs and cheese and a high-glucose drink (1.1 g glucose.kg body weight^-1^ dissolved in 200 ml of water). The high-glucose drink contained on average 87.6 ± 10.4 g of pure glucose (a range of 76 – 106 g) and was therefore similar to, or in most cases above that of a standard oral glucose tolerance test (75 g glucose). Participants were instructed to consume the drink within 1 min and the meal within 5 min. Venous blood samples were taken every 30 min over the 2-h postprandial period.

### 2.4 Exercise trial (3 h and 24 h post-exercise meals)

Participants arrived in the laboratory after an overnight fast and an intravenous cannula was inserted into an antecubital fossa vein. After 30 minutes of rest on a bed, a baseline blood sample was taken. Participants then underwent the acute exercise session which involved 1 h of cycling at 70-75% VO_2peak_ with an additional 3-min warm-up and cool-down period at 50% VO_2peak_. After completion of the exercise session participants rested for 3 h on the bed and then underwent the 2-h high-glucose mixed-nutrient meal challenge (as previously described) to assess postprandial responses at 3 h post-exercise. The following day, participants arrived in the laboratory after an overnight fast and underwent the same 2-h high-glucose mixed-nutrient meal challenge to assess postprandial responses at 24 h post-exercise. Venous blood samples were collected before and immediately after exercise, prior to meal ingestion (3 h and 24 h post-exercise), and every 30 mins throughout the 2-h postprandial period.

### 2.5 Redox homeostasis analyses

Venous blood was allowed to clot at room temperature in a Vacutainer^®^ serum separating tube (SST) for 20 min. Venous blood was also collected in an ethylenediaminetetraacetic acid (EDTA) Vacutainer^®^ tube and placed immediately on ice. EDTA and SST tubes were separated by centrifugation (10 min at 1800 Relative Centrifugal Force, 4°C) and immediately aliquoted and stored at -80°C until analysis. Optimization of assays revealed an optimal sample dilution factor of 2x for catalase activity, whereas all other assays did not require sample dilution. All assay plates were read using a spectrophotometer plate reader (Synergy H1, BioTek, USA) and all samples were measured in duplicate. Average inter-assay coefficient of variation (CV) was determined by calculating the CV for the standards between each 96 well plate. Average intra-assay CV was determined by calculating the CV between each duplicate and then averaging the CV across all samples.

### 2.6 Plasma analysis

Commercial assay kits were used to measure SOD (Cayman Chemical, USA; Catalogue Number 706002) and H_2_O_2_ (Thermo Fisher Scientific, USA; Catalogue Number A22188) in plasma, as per manufacturer instructions. An inter-assay CV of 10.87% and 1.42% and intra-assay CV of 5.42% and 1.24% were calculated for SOD and H_2_O_2_, respectively.

### 2.7 Serum analysis

Commercial assay kits were used to measure catalase activity (Cayman Chemical, USA; Catalogue Number 707002), 8-isoprostanes (RayBitotech Inc, USA; Catalogue Number EIA-IPF2A), TBARS (Cayman Chemical, USA; Item No. 700870), and nitrate+nitrite concentrations ([NOx] Cayman Chemical, USA; Item No. 780001) in serum. An inter-assay CV of 6.95% and intra-assay CV of 5.25% was calculated for catalase activity. An inter-assay CV of 7.40% and intra-assay CV of 2.75% was calculated for 8-isoprostanes. An inter-assay CV of 4.79% and intra-assay CV of 2.59% was calculated for TBARS. An inter-assay CV of 2.71% and intra-assay CV of 1.38% was calculated for NOx (nitrate+nitrite), and an inter-assay CV of 1.90% and intra-assay CV of 1.00% was calculated for nitrite alone. Nitrate was calculated as NOx (nitrate+nitrite) minus nitrite, as per the manufacturer’s instructions.

### 2.8 Statistical analysis

Data were checked for normality using Shapiro–Wilk normality tests. Catalase, H_2_O_2_, TBARS, 8-isoprostanes, and nitrite were not normally distributed and therefore log transformed using the natural log prior to statistical analysis. Exercise only data (baseline, immediately post-exercise and 3 h post-exercise prior to meal ingestion) were analyzed using a one-way ANOVA with “time” as the within-subjects factor. Postprandial data were analyzed using a two-way repeated measures ANOVA with “time” (pre-meal and postprandial timepoints) and “condition” (control meal, 3 h post-exercise meal, and the 24 h post-exercise meal) as the within-subjects factors. *A priori* comparisons between baseline (pre-meal) and each postprandial timepoint, and between conditions at the same timepoint, were conducted using Fishers Least Significant Difference test when there was evidence for “main” and “interaction” effects at the p ≤ 0.05 level. Individual p-values are reported to provide the level of confidence in reported effects. To graphically represent individual responses, the delta change is reported for post-exercise responses and the incremental area under the curve (iAUC) is reported for postprandial responses using the trapezoidal rule. Column graphs are presented as Box and Whisker plots with the Median, Mean, and Interquartile Range. Line graphs are presented as mean ± SEM to preserve clarity. One participant had insufficient sample quality for all timepoints in the 24 h post-exercise trial for NOx measurements (nitrate+nitrite, nitrate, and nitrite). These data points were excluded from analysis after confirming statistical outlier status *via* Extreme Studentized Deviate test (all p < 0.05). A mixed-effects model was used to statistically analyze NOx measurements due to the missing values.

## 3 Results

Postprandial glucose and insulin responses to the control meal, and 3 h and 24 h post-exercise meals, are reported in a previous publication (41).

### 3.1 The effect of acute exercise on systemic markers of redox homeostasis

**Figure 1:**
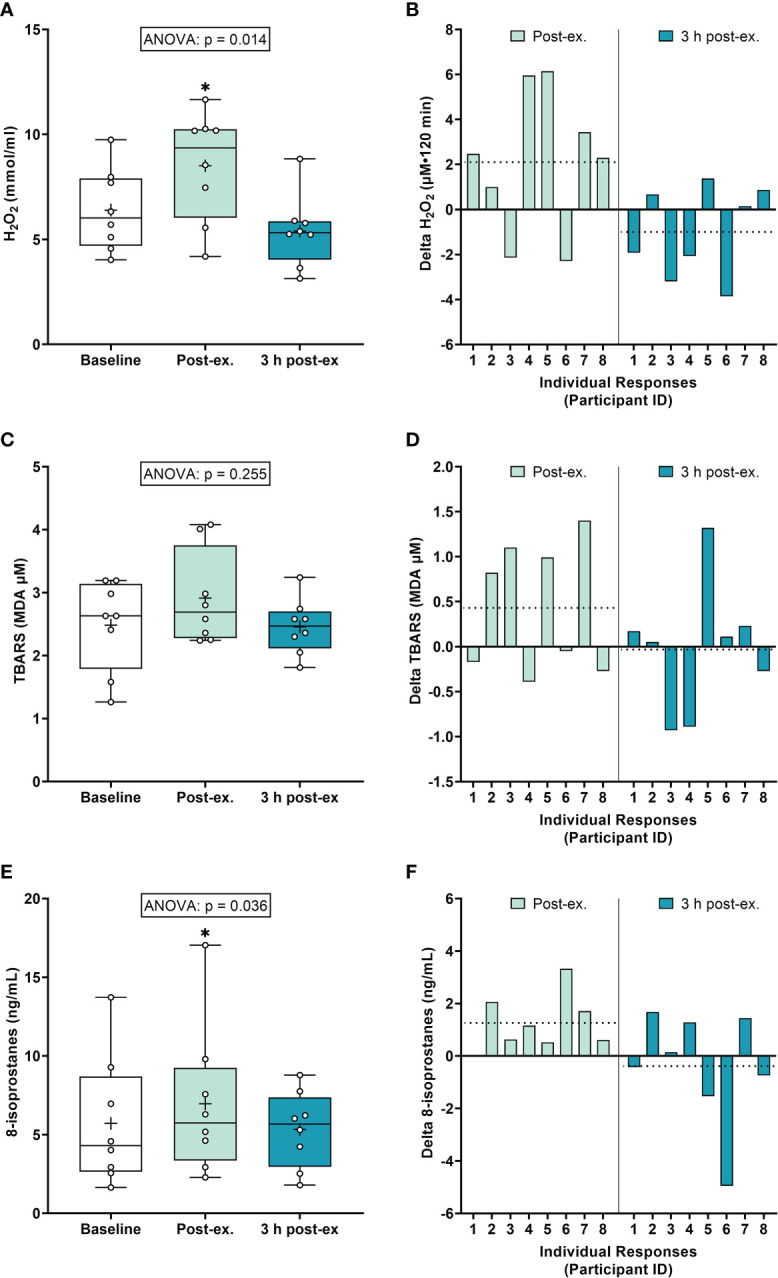
Plasma hydrogen peroxide (H_2_O_2_) **(A, B)**, serum thiobarbituric acid reactive substances (TBARS) **(C, D)**, and 8-isoprostanes **(E, F)** at baseline, immediately after exercise, and 3 h post-exercise. Group averages and individual data **(A, C, E)** are presented as Box and Whisker plots. The Box represents the interquartile range alongside the median (line) and mean (plus symbol). The Whiskers represent the minimum and maximum range of the data. Individual responses **(B, D, F)** are presented as the delta change between baseline and post-exercise timepoints (immediately post-exercise and 3 h post-exercise). The dotted line represents the group average delta value. Data were analyzed using one-way ANOVA and Fishers LSD test for multiple comparisons. n = 8 participants. *p < 0.05 compared to baseline.

*Oxidative stress markers.* On average (group means), H_2_O_2_ increased immediately after exercise compared to the pre-exercise baseline (p = 0.040), and then returned to near baseline levels at 3 h post-exercise (p = 0.300; **Figures 1A, B**). Average TBARS levels were not altered immediately after exercise or 3 h post-exercise (ANOVA p = 0.255; **Figures 1C, D**). Average 8-isoprostane levels increased immediately after exercise compared to the pre-exercise baseline (p = 0.018), and then returned to near baseline levels at 3 h post-exercise (p = 0.726; **Figures 1E, F**).

**Figure 2:**
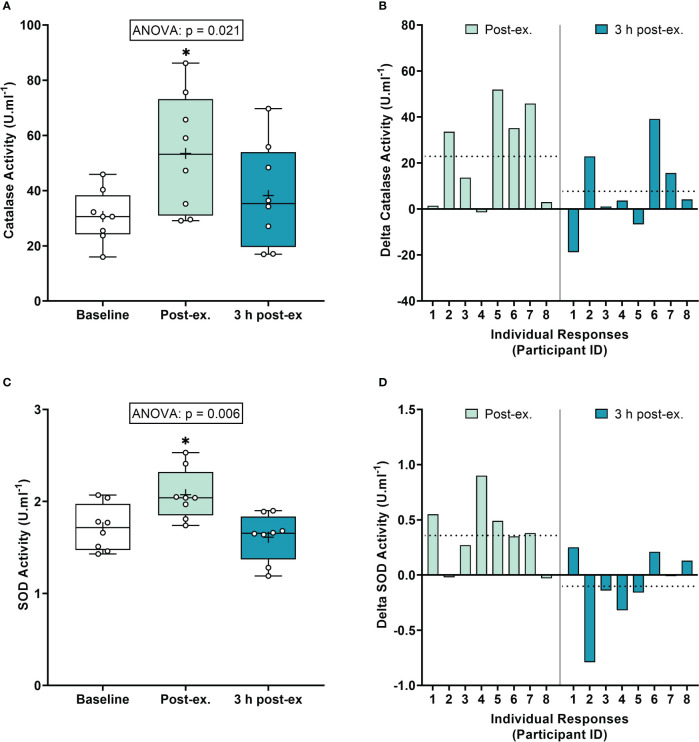
Serum catalase activity **(A, B)** and plasma superoxide (SOD) activity **(C, D)** at baseline, immediately after exercise, and 3 h post-exercise. Group averages and individual data **(A, C)** are presented as Box and Whisker plots. The Box represents the interquartile range alongside the median (line) and mean (plus symbol). The Whiskers represent the minimum and maximum range of the data. Individual responses **(B, D)** are presented as the delta change between baseline and post-exercise timepoints (immediately post-exercise and 3 h post-exercise). The dotted line represents the group average delta value. Data were analyzed using one-way ANOVA and Fishers LSD test for multiple comparisons. n = 8 participants. *p < 0.05 compared to baseline.

*Antioxidant defense markers.* Average catalase activity levels increased immediately after exercise compared to the pre-exercise baseline (p = 0.008), and then returned to near baseline levels at 3 h post-exercise (p = 0.377; **Figures 2A, B**). Average SOD activity levels increased immediately after exercise compared to the pre-exercise baseline (p = 0.013), and then returned to near baseline levels at 3 h post-exercise (p = 0.425; **Figures 2C, D**).

**Figure 3:**
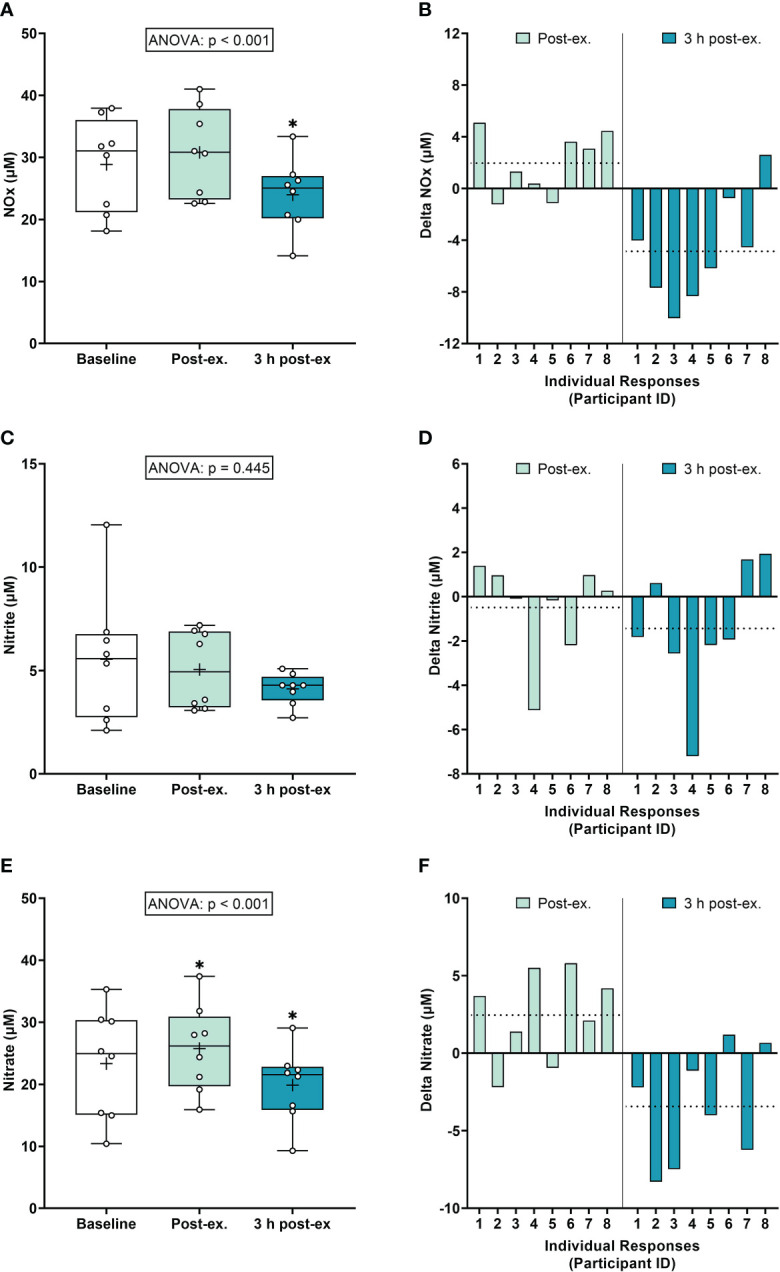
Serum Nitrate+Nitrite (NOx) **(A, B)**, Nitrite **(C, D)**, and Nitrate **(E, F)** at baseline, immediately after exercise, and 3 h post-exercise. Group averages and individual data **(A, C, E)** are presented as Box and Whisker plots. The Box represents the interquartile range alongside the median (line) and mean (plus symbol). The Whiskers represent the minimum and maximum range of the data. Individual responses **(B, D, F)** are presented as the delta change between baseline and post-exercise timepoints (immediately post-exercise and 3 h post-exercise). The dotted line represents the group average delta value. Data were analyzed using one-way ANOVA and Fishers LSD test for multiple comparisons. n = 8 participants. *p < 0.05 compared to baseline.

*NOx markers.* Average NOx levels (Nitrate+Nitrite) remained similar to pre-exercise baseline levels immediately after exercise (p = 0.118), and then decreased below baseline levels 3 h post-exercise (p < 0.001; **Figures 3A, B**). Average nitrite levels remained similar to baseline levels immediately post-exercise and 3 h post-exercise (ANOVA, p = 0.445; **Figures 3C, D**). Average nitrate levels increased immediately after exercise compared to the pre-exercise baseline (p = 0.035), and then decreased below baseline levels at 3 h post-exercise (p = 0.005; **Figures 3E, F**).

*Inter-individual exercise responses.* Observation of individual responses indicated that exercise-induced oxidative stress, antioxidant activity, and NOx levels, were highly variable at the individual level and depended on the biomarker and post-exercise timepoint investigated (**Figures 1B, D, F**, **2B, D**, **3B, D, F**).

### 3.2 The effects of high-glucose ingestion and prior exercise on systemic markers of redox homeostasis

#### 3.2.1 Oxidative stress markers

**Figure 4:**
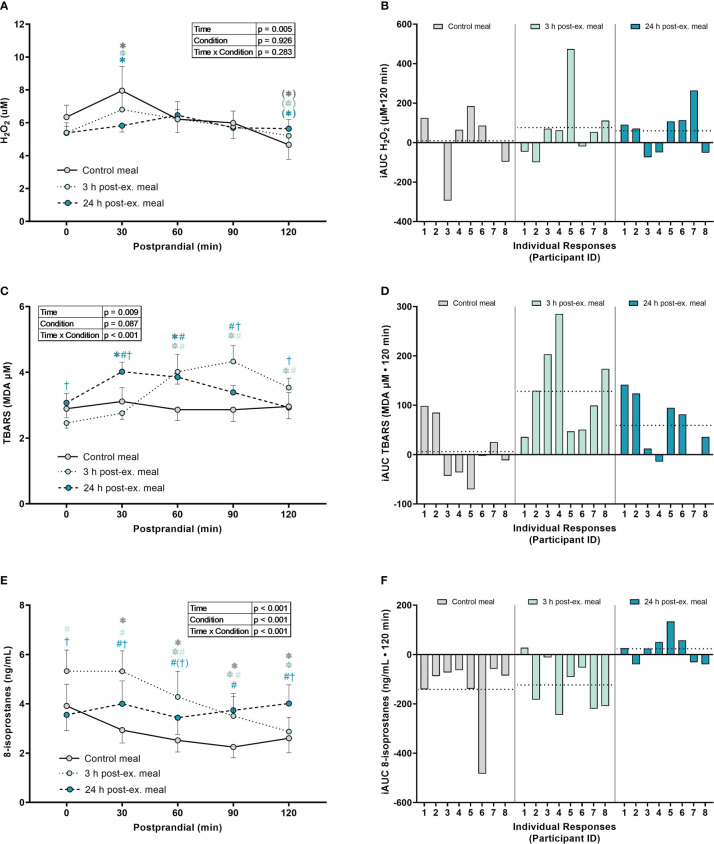
The effects of a high-glucose mixed nutrient meal on plasma hydrogen peroxide (H_2_O_2_) **(A, B)**, serum thiobarbituric acid reactive substances (TBARS) **(C, D)**, and 8-Isoprostanes **(E, F)**, when ingested at rest (no-exercise control meal), and 3 h and 24 h post-exercise. Line graphs **(A, C, E)** are presented as mean ± SEM. Individual responses **(B, D, F)** are presented as the incremental area under the curves (iAUC) following meal ingestion at rest (control meal), and 3 h and 24 h post-exercise. The dotted line represents the group average iAUC value. Postprandial data (line graphs) were analyzed using a two-way ANOVA with “time” (pre-meal and postprandial timepoints) and “condition” (control meal, 3 h post-exercise meal, and 24 h post-exercise meal) as the within-subjects factors. Fishers LSD test was used for multiple comparisons. *p < 0.05 compared to pre-meal levels (0-min). ^#^p < 0.05 compared to the same timepoint in the control condition. ^†^p < 0.05 compared to the same timepoint in the 3 h post-exercise condition. Symbols in parenthesis indicate p < 0.1. n = 8 participants.

*Hydrogen peroxide.* A main effect of time was detected for average H_2_O_2_ levels (p = 0.005; **Figures 4A, B**). Compared to pre-meal levels (0-min), average H_2_O_2_ levels increased at 30 min postprandial (p = 0.050) and then decreased below pre-meal levels at 120 min postprandial (p = 0.060), regardless of when the meal was ingested. No interaction (p = 0.283) or condition effects were detected (p = 0.926).

*TBARS.* An interaction effect was detected for TBARS (p < 0.001; **Figures 4C, D**). Compared to pre-meal levels, average TBARS levels were elevated between 60 min and 120 min in the 3 h post-exercise condition (all p ≤ 0.001) and were elevated at 30 min and 60 min postprandial in the 24 h post-exercise condition (all p < 0.018). In contrast, TBARS remained similar to pre-meal levels throughout the 2-h postprandial period in the control condition (all p ≥ 0.632). Comparison between conditions indicated that TBARS was elevated in the 3 h post-exercise condition compared to the control condition at 60 min (p = 0.003), 90 min (p < 0.001) and 120 min (p = 0.029) postprandial. TBARS in the 3 h post-exercise condition was also lower pre-meal (p = 0.044) and 30 min (p < 0.001) postprandial, and elevated at 90 min (p = 0.035) and 120 min (p = 0.049) postprandial when compared to the 24 h post-exercise condition. Compared to the control condition, TBARS in the 24 h post-exercise condition was elevated at 30 min (p = 0.004), 60 min (p = 0.002) and 90 min (p = 0.039) postprandial.

*8-isoprostanes.* An interaction effect was detected for 8-isoprostane levels (p < 0.001; **Figures 4E, F**). Compared to pre-meal levels, average 8-isoprostane levels were lower at 30 min postprandial (p = 0.009) in the control condition, and lower between 60 min and 120 min postprandial in both the control and 3 h post-exercise conditions (all p ≤ 0.005). In contrast, 8-isoprostanes in the 24 h post-exercise condition remained similar to pre-meal levels throughout the 2-h postprandial period (all p ≥ 0.218). Comparison between conditions revealed that 8-isoprostane levels were higher in the 3 h post-exercise condition at baseline, 30 min, 60 min, and 90 min postprandial compared to the control condition (all p < 0.001). 8-isoprostane levels in the 3 h post-exercise condition were also higher pre-meal (p < 0.001), 30 min (p < 0.001), and 60 min (p = 0.058) postprandial, and lower 120 min postprandial (p < 0.001), when compared to the 24 h post-exercise condition. 8-isoprostane levels were higher between 30 and 120 min postprandial in the 24 h post-exercise condition compared to the control condition (all p ≤ 0.013).

#### 3.2.2 Antioxidant defense markers

**Figure 5:**
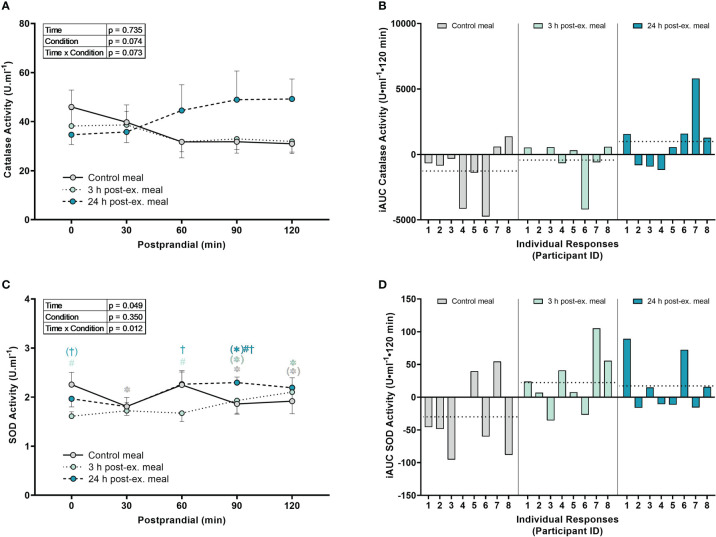
The effects of a high-glucose mixed nutrient meal on serum catalase activity **(A, B)** and plasma superoxide activity (SOD) activity **(C, D)** when ingested at rest (no-exercise control meal), and 3 h and 24 h post-exercise. Line graphs **(A, C)** are presented as mean ± SEM. Individual responses **(B, D)** are presented as the incremental area under the curves (iAUC) following meal ingestion at rest (control meal), and 3 h and 24 h post-exercise. The dotted line represents the group average iAUC value. Postprandial data (line graphs) were analyzed using a two-way ANOVA with “time” (pre-meal and postprandial timepoints) and “condition” (control meal, 3 h post-exercise meal, and 24 h post-exercise meal) as the within-subjects factors. Fishers LSD test was used for multiple comparisons. *p < 0.05 compared to pre-meal levels (0-min). ^#^p < 0.05 compared to the same timepoint in the control condition. ^†^p < 0.05 compared to the same timepoint in the 3 h post-exercise condition. Symbols in parenthesis indicate p < 0.1. n = 8 participants.

*Catalase activity.* There were no interaction (p = 0.073), condition (p = 0.074), or time effects (p = 0.735) for average catalase activity (**Figures 5A, B**).

*SOD activity.* An interaction effect was detected for SOD activity (p = 0.012; **Figures 5C, D**). Compared to pre-meal levels, average SOD activity levels were lower at 30 min (p = 0.015), 90 min (p = 0.032) and 120 min postprandial in the control condition (p = 0.063), higher at 90 min (p = 0.079) and 120 min postprandial (p = 0.008) in the 3 h post-exercise condition, and higher at 90 min postprandial (p = 0.072) in the 24 h post-exercise condition. Comparison between conditions revealed that SOD activity in the 3 h post-exercise condition was lower pre-meal (p < 0.001) and 60 min (p = 0.002) postprandial compared to the control condition, and lower pre-meal (p = 0.051), 60 min (p = 0.002), and 90 min postprandial (p = 0.047) compared to the 24 h post-exercise condition. SOD activity was lower at 90 min postprandial in the 24 h post-exercise condition compared to the control condition (p = 0.019).

#### 3.2.3 NOx markers

**Figure 6:**
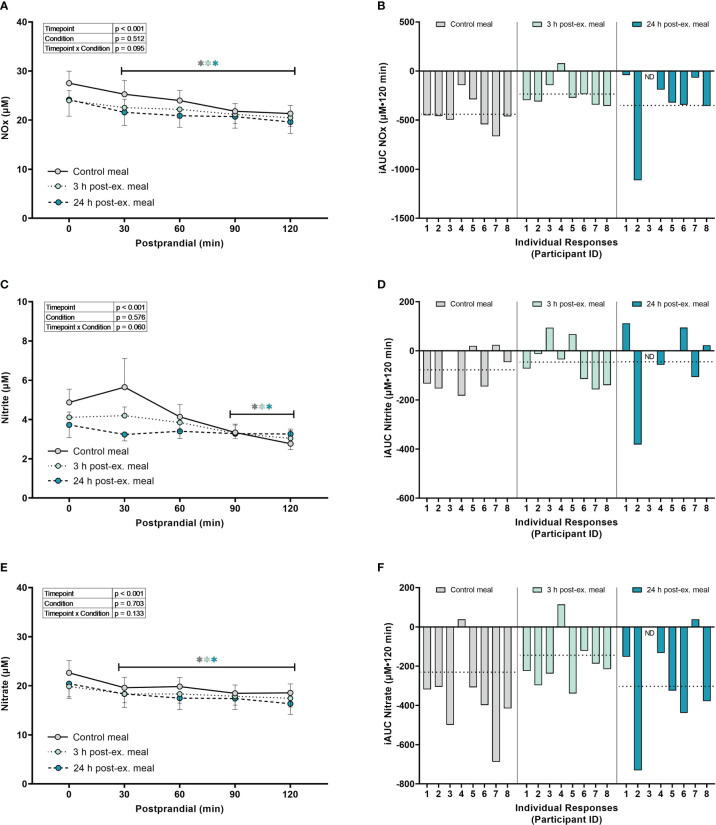
The effects of a high-glucose mixed nutrient meal on serum Nitrate+Nitrite (NOx) **(A, B)**, Nitrite **(C, D)**, and Nitrate **(E, F)**, when ingested at rest (no-exercise control meal), and 3 h and 24 h post-exercise. Line graphs **(A, C, E)** are presented as mean ± SEM. Individual responses **(B, D, F)** are presented as the incremental area under the curves (iAUC) following meal ingestion at rest (control meal), and 3 h and 24 h post-exercise. The dotted line represents the group average iAUC value. Postprandial data (line graphs) were analyzed using a mixed-effects model with “time” (pre-meal and postprandial timepoints) and “condition” (control meal, 3 h post-exercise meal, and 24 h post-exercise meal) as the within-subjects factors. Fishers LSD test was used for multiple comparisons. *p < 0.05 compared to pre-meal levels (0-min). n = 8 participants for control and 3 h post-exercise meals, and n = 7 for the 24 h post-exercise meal. ND, no data due to insufficient sample quality. Symbols in gray represent statistical outcomes for the control meal, symbols in light green represent statistical outcomes for the 3 h post-ex meal and symbols in blue represent statistical outcomes for the 24 h post-ex meal.

*Combined nitrate+nitrite (NOx).* A main effect of time (p = 0.003) was detected for NOx (p < 0.001). Compared to pre-meal levels, average NOx levels decreased at 30 min (p = 0.002) postprandial and remained lower than pre-meal levels throughout the 2-h postprandial period (all p < 0.001; **Figures 6A, B**) regardless of condition. There were no condition (p = 0.512) or interaction effects for NOx (p = 0.095).

*Nitrite.* A main effect of time (p < 0.001) was detected for nitrite (**Figures 6C, D**). Compared to pre-meal levels, average nitrite levels decreased at 90 min (p = 0.002) and remained lower at 120 min postprandial (p < 0.001) regardless of condition. There were no condition (p = 0.576) or interaction effects for nitrite (p = 0.060).

*Nitrate.* A main effect of time (p < 0.001) was detected for nitrate (**Figures 6E, F**). Compared to pre-meal levels, nitrate levels decreased at 30 min postprandial (p < 0.001) and remained lower than pre-meal levels throughout the 2-hour postprandial period (all p < 0.001) regardless of condition. There were no condition (p = 0.703) or interaction effects for nitrate (p = 0.133).

*Inter-individual postprandial responses.* Similar to exercise, observation of individual responses indicated that postprandial oxidative stress, antioxidant activity, and NOx levels, were highly variable at the individual level and depended on the biomarker, postprandial timepoint, and whether the meal was ingested after exercise (**Figures 4B, D, F**, **5B, D**, **6B, D, F**).

## 4 Discussion

We provide evidence that systemic redox homeostasis is complex and dynamically altered following acute exercise, high-glucose ingestion, and when exercise is performed prior to high-glucose ingestion. In support of the general academic consensus (13, 14, 28), acute exercise increased several markers of oxidative stress and antioxidant activity (H_2_O_2_, 8-isoprostanes, catalase, SOD, and nitrate). However, not all markers were influenced in a consistent manner, and all markers exhibited large inter-individual variability in the magnitude and/or direction of responses. High-glucose ingestion at rest, and when ingested 3 h and 24 h after exercise, also led to alterations in redox homeostasis as indicated by changes in TBARS, catalase, SOD, H_2_O_2_, 8-isoprostanes, and NOx activity. Similar to acute exercise, postprandial responses exhibited large individual responsiveness and were dynamically influenced by factors including the timing of the meal (rest, 3 h and 24 h post-exercise), the postprandial time-point, and the specific biomarker measured. These findings highlight the necessity to interpret redox responses in the context of the individual, biomarker measured, and stimuli observed (e.g., exercise, high-glucose ingestion, or high-glucose ingestion after exercise). Findings may suggest the existence of individual redox responsiveness. This individual variability may be of physiological relevance and should be investigated in future studies as a means to inform personalized redox interventions that target both oxidative distress and eustress (6, 31).

### 4.1 The effect of acute exercise on markers of systemic redox homeostasis

Increased ROS production caused by skeletal muscle contraction during exercise (1, 42–46) may be beneficial and necessary for cell function including glucose cell transport (47, 48); induction of endogenous antioxidant enzymes (44, 49, 50); mitochondrial biogenesis (16, 18, 51); muscle contraction force production (52–54); and vascular health and function (55–58). As such, exploring the effect of exercise on redox homeostasis has become paramount to identifying effective strategies for health promotion and the treatment and prevention of disease. However, directly measuring ROS in human tissue is methodologically difficult, which has led to a heavy reliance on the measurement of indirect markers of oxidative stress derived from whole tissue analysis such as blood or homogenized skeletal muscle (59, 60). Our findings show that H_2_O_2_, 8-isoprostanes, catalase, SOD, and nitrate levels measured in serum/plasma are transiently increased after a single session of aerobic exercise. These findings support previous reports that systemic oxidative stress and antioxidant activity can be altered (increased or decreased) by exercise (12, 15–18, 29, 61, 62). Interestingly, in the current study we observed that at the group level, many markers, such as H_2_O_2_ and nitric oxide markers, return to baseline levels at 3 hours post exercise. This decrease after 3 hours could be a result of increased ROS post exercise causing a removal or decrease in the bioavailability of NOx and other antioxidant markers. Future studies should employ more frequent time points to further investigate at what point this decrease occurs.

Not all studies, however, have reported consistent changes within and between different measures of redox homeostasis after exercise (11, 28–30, 63). For example, one study reported that TBARS in untrained adults is elevated at 5 and 10 mins post-exercise, returning to near baseline levels at 20 and 30 mins post-exercise (63), suggesting an immediate and transient effect of exercise on systemic oxidative stress. In contrast, others have reported that TBARS peak 1 hour post-exercise in untrained individuals (29), whereas in a previous study we reported a decrease in TBARS following acute aerobic exercise in heathy young and obese middle-aged adults (11, 30). In the present study, TBARS was largely unchanged after acute exercise, at least when observed at the group level. These conflicting reports continue across the spectrum of different biomarkers used when measuring system redox homeostasis in humans (28). A number of experimental and methodological factors such as the timing of post-exercise measurements, the population investigated, the type of exercise intensity, volume and mode, and the large variety of biomarkers assessed (13, 14, 29, 30, 59, 60, 64–66), are likely to contribute to the contradictory findings among studies. Although less well studied, the concept of interindividual variability, or in other words an individual’s “redox phenotype”, may also be contributing to the large range of responses reported in the literature (31, 32). This is not surprising given the evidence of high and low responders to exercise training and the individual responsiveness reported in adaptation to different modalities and intensities of exercise training, across a broad range of physiological variables (33–36).

Research by Margaritelis, Kyparos (31) revealed a large inter-individual redox variability in response to isokinetic eccentric exercise, in which individuals (beyond that observed at the group level) increased, decreased or exhibited negligible change post-exercise in urinary F2-isoprostanes, plasma protein carbonyls, and erythrocyte glutathione levels. Subsequent research confirmed this phenomenon even after adjusting for the regression to the mean artifact (32). Likewise, Margaritelis, Theodorou (6) established that healthy individuals could be stratified into low, moderate, and high-level responders with respect to exercise-induced oxidative eustress (6), despite investigating a seemingly homogonous population of recreationally active young male individuals matched for common physiological parameters (e.g., age, weight, body fat percentage, dietary intake). This concept of individual responsiveness, and thus the requirement for personalized intervention, has also been extended to antioxidant treatment in humans (13, 39). Although limited in sample size, a potential example of individual responsiveness was observed with TBARS in the current study, where four participants exhibited increased TBARS levels following acute exercise and four individuals exhibited either a decrease or negligible change. Other markers such as catalase and 8-isoprostanes also exhibited potential inter-individual variations in the magnitude of the post-exercise response, observations that may have otherwise been masked if only reporting group responses. Our findings support the requirement to report individual responses alongside group means and statistics. Equally important is the future exploration of potential mechanisms that dictate certain redox phenotypes, and the physiological relevance of individual redox responsiveness in the context of both health and disease. Given that exercise-induced oxidative eustress has been linked to exercise-induced training health adaptations (exercise capacity and performance) (6), finding a way to personalize or optimize the oxidative eustress response at the individual level would be a worthwhile endeavor.

### 4.2 The effect of high-glucose mixed-nutrient meal ingestion on markers of systemic redox homeostasis

Alterations in postprandial oxidative stress are commonly reported following the ingestion of a range of meals varying in macronutrient composition (e.g., high-fat, high-glucose, protein only, and mixed nutrient meals), size (e.g., high-calorie versus lower calorie), and type (liquid meal versus solid meals) (66–79). The current study also found an altered state of systemic redox homeostasis following high-glucose mixed nutrient meal ingestion as indicated by a decrease in postprandial SOD, 8-isoprostanes and NOx measures, and increase in H_2_O_2_. In contrast, TBARS and catalase activity remained similar to baseline throughout the postprandial period.

The observed lack of increase in postprandial TBARS is somewhat surprising, as we previously reported increased TBARS in overweight and obese individuals 1 hour after ingesting a breakfast containing cereal, full-cream milk, and honey (66). However, others have also reported a lack of change in the lipid peroxidation marker of malondialdehyde in heathy adults following an OGTT (75 g glucose) (80), dextrose (81), and maltodextrin ingestion (2.25 g/kg) (81). The lack of increase in TBARS following meal ingestion in the current study may be due to presumably normal glucose tolerance in healthy individuals, in-part supported by reports that TBARS increases following an OGTT in participants with impaired glucose tolerance but not in healthy controls (82). Postprandial oxidative stress may also be influenced by other factors such as the size and composition of the meal (high-lipid versus carbohydrate/mixed nutrient meals) (78, 79), potential sex differences (75), and what could be individual redox responsiveness to the meal.

In the present study, five participants exhibited elevated TBARS 30 min postprandial, whereas three participants exhibited decreased levels at the same timepoint. Other biomarkers such as catalase, H_2_O_2_, and SOD activity exhibited similar individual variability in the magnitude and direction of responses. Few studies have reported individual responses which hamper the interpretation of previous studies. For example, Kawano, Motoyama (83), Xiang, Sun (82) and Fisher-Wellman and Bloomer (78) reported no change in TBARS or MDA following high-carbohydrate ingestion (OGTT or Dextrose drink) in healthy individuals. However, a postprandial trend was reported in one study (83) and in all studies group mean postprandial values were elevated which may be indicative of individual responsiveness. Future studies would benefit from reporting individual responses alongside group statistics to help facilitate data interpretation and the understanding of individualized responsiveness in human redox homeostasis (6) (31). Margaritelis, Paschalis (39) reported improved exercise capacity and performance in healthy individuals following 30 days of antioxidant treatment only when the treatment was personalized by targeting specific antioxidant deficiencies at the individual level. Our findings support that a similar personalized redox-intervention approach may be required to effectively target glycemia-related oxidative distress.

### 4.3 Prior exercise and postprandial oxidative stress

Acute exercise has the capacity to transiently increase antioxidant activity (11, 29, 65, 70) and can improve insulin sensitivity and whole-body glucose uptake for up to 24-48 h post-exercise (11, 14, 66, 84–89). As such, exercise prior to meal ingestion may be a potential method to decrease postprandial oxidative distress and to mitigate the health complications associated with elevated blood glucose and lipid levels (9, 14, 70). In some situations, acute exercise has been reported to attenuate postprandial oxidative stress (66, 70, 71, 76, 90), whereas in other situations this has not been the case (66, 69, 71, 91, 92). Similarly, in the present study we report conflicting results where postprandial oxidative stress was either unchanged (H_2_O_2_) or elevated (TBARS and 8-isoprostanes) after acute exercise when compared to control meal levels.

The reason for elevated postprandial TBARS and 8-isoprostanes after exercise are unknown, but may be related to the increase in postprandial blood glucose levels that are commonly reported when glucose is ingested in the hours after acute exercise (41, 88, 89, 93). In this same cohort of individuals, we previously reported elevated postprandial glucose levels when the high-glucose mixed nutrient meal was ingested 3 h post-exercise (41), a finding which likely reflects increased glucose release from splanchnic tissue to prioritize skeletal muscle glycogen replenishment (93). Kawano, Motoyama (83) reported a similar observation between elevated postprandial blood glucose levels and TBARS. We also previously observed greater postprandial oxidative stress alongside elevated glucose levels when high-intensity exercise was performed 1 hour after ingesting a standard breakfast containing cereal, milk, and honey (66). Of note, Bloomer, Kabir (79) reported similar postprandial glucose levels, and subsequently similar levels of oxidative stress, when comparing 75 g and 150 g of dextrose ingestion in young healthy adults. Taken together, our findings and others suggest that postprandial oxidative stress may be closely associated with the postprandial glucose excursion observed, as opposed to the absolute load of glucose ingested. This is an interesting phenomenon given that numerous factors, such as health status (e.g., excess adiposity and insulin resistance) and exercise (e.g., acute exercise and exercise training) can influence postprandial glucose responses. The physiological relevance of glucose-induced oxidative stress under these different conditions of hyperglycemia (or elevated glucose levels) remains to be elucidated.

ROS production following sustained levels of elevated blood glucose and lipid levels can lead to a state of oxidative distress which is reported to contribute to various acute and chronic health complications including vascular dysfunction and impaired glycemic control (5, 20, 73, 94, 95). However, in many cases ROS are beneficial for health and required for normal substrate oxidation and vascular function (2–4, 10, 20, 55, 96–100). Indeed, in the same cohort of individuals as the current study, we reported improved microvascular blood flow in skeletal muscle when the meal was ingested after exercise (41), which occurred despite the presence of elevated postprandial oxidative stress. Other studies have also reported improved whole-body glucose disposal following acute exercise despite elevated glucose levels (11, 88, 89). As such, evidence suggests that the well-established link between elevated glucose, postprandial oxidative stress, and impaired insulin sensitivity may be unreliable in the context of exercise, and as such, elevated postprandial oxidative stress after exercise may simply reflect a combination of both oxidative eustress and distress. The contradictory nature of measuring systemic markers following complex stimuli is not surprising given the growing emphasis that redox regulation of health and disease is likely to be dictated by compartmentalized redox reactions and redox cell signaling that occur at the subcellular level (13, 59, 101); complex cell physiology that is unlikely to be resolved by systemic biomarkers assessed in whole tissue samples (i.e., blood or homogenized muscle). Our findings support that systemic markers of redox homeostasis in serum/plasma may lack the physiological resolution to differentiate between oxidative eustress or distress in humans during complex and dynamic physiological scenarios (e.g., meal ingestion after exercise). Future studies characterizing the redox response to stimuli using more sophisticated techniques in redox biology, such as redox proteomics, would prove to be beneficial and help pave the way for future personalized redox intervention.

### 4.4 Limitations

Redox biomarkers were analyzed in plasma/serum samples derived from venous blood. Arteriovenous differences in glycaemia have been reported following high-glucose ingestion in healthy men which can be altered by prior exercise (102). Arteriovenous differences in redox biomarkers are seldom investigated, with one study reporting variable responses at rest and after exercise (103), although findings were restricted by sample size (n = 4). Future studies are required to investigate potential arteriovenous redox differences in biomarkers during dynamic metabolic conditions such as following exercise and meal consumption in humans. Additionally, commercial assay kits used to analyze oxidative stress biomarkers in this study are not without limitations. Biomarkers of oxidative stress measured in blood or muscle tissue are commonly used to study redox stimuli and identify conditions of oxidative distress (e.g., glycemia-induced oxidative stress) or oxidative eustress (e.g., exercise-induced oxidative stress) (13, 14, 28). However, these measures of redox homeostasis in blood or muscle tissue samples are unlikely to reflect more complex compartmentalized redox reactions and cell signaling that occurs at the subcellular level, which in part, may dictate whether a redox stimulus is beneficial (oxidative eustress) or detrimental (oxidative distress) (1, 13, 59, 101, 104, 105). This provides a challenge to researchers as certain redox stimuli, for example substrate metabolism and muscle contraction/exercise, are likely to elicit varying degrees of oxidative eustress and/or distress at the cellular level (3, 4, 7, 20, 52, 95, 104, 106, 107), which may not be reflected at the systemic level. This issue is relevant to all oxidative stress biomarkers, even those regarded as the gold standard (e.g., isoprostanes). Future studies are required to confirm and link systemic measures of oxidative stress/antioxidant activity to that of functional health outcomes (such as glycemic control) in humans.

Another limitation is that redox responses to the 24 h post-exercise meal may have been influenced by the preceding glucose ingestion 3 h post-exercise. Substantial glycogen replenishment occurs when carbohydrates (100 g) are ingested in the hours following exercise which can influence insulin sensitivity and glucose responses to subsequent carbohydrate ingestion the following day (108). Further investigation of how nutrient composition and post-exercise meal timing influences postprandial redox biomarkers is warranted.

The majority of research investigating redox responses to redox stimuli have only reported group statistics. This has led to a lack of understanding around the potential existence of individual redox responsiveness. A strength of this manuscript is that we have presented data as both the group mean and individual data points. The aim of reporting individual data points is to bring to light the importance of investigating such responses and highlight the large variability in redox responses following acute exercise, high-glucose meal ingestion, and high-glucose meal ingestion after exercise. Although individual redox responsiveness to exercise has been shown before (32), future research will be required using a more appropriate methodological approach to confirm whether the observed variation is truly an indication of individual responsiveness (109).

## 5 Conclusion

Our data support the notion of individual redox responsiveness to exercise, meal consumption, and meal consumption after exercise; findings which not only highlight the importance of reporting individual data, but also hint at a future in which personalized redox interventions are investigated for targeting both oxidative eustress and distress. We also demonstrate the inability of systemic markers to reliably reflect a state of oxidative eustress or distress in response to more complex redox stimuli (e.g., meal ingestion after acute exercise) which likely requires targeted analysis of compartmentalized redox reactions that occur at the subcellular level. The measurement of redox homeostasis in humans at the systemic level is undoubtably useful to inform upon overall health and/or disease status including the identification of elevated oxidative stress and antioxidant deficiencies (13, 39, 40). However, our findings highlight the necessity to interpret redox biomarkers in the context of the individual, biomarker measured, and stimuli observed.

## Data availability statement

The raw data supporting the conclusions of this article will be made available by the authors, without undue reservation.

## Ethics statement

The studies involving human participants were reviewed and approved by The Deakin University Human Research Ethics Committee. The patients/participants provided their written informed consent to participate in this study.

## Author contributions

HT: Conceptualization, Data Curation, Writing - Original Draft, Writing - Review & Editing. TA: Investigation, Data Curation, Writing - Review & Editing. DM: Methodology, Investigation, Data Curation, Writing - Review & Editing. MK: Conceptualization, Methodology, Investigation, Data Curation, Writing - Review & Editing, Supervision. LP: Conceptualization, Methodology, Investigation, Data Curation, Writing - Original Draft, Writing - Review & Editing, Supervision, Project Administration, Funding Acquisition. All authors contributed to the article and approved the submitted version.
